# A suspended act: increased reflectivity and gender-dependent electrophysiological change following Quadrato Motor Training

**DOI:** 10.3389/fpsyg.2014.00055

**Published:** 2014-02-03

**Authors:** Tal Dotan Ben-Soussan, Aviva Berkovich-Ohana, Joseph Glicksohn, Abraham Goldstein

**Affiliations:** ^1^The Leslie and Susan Gonda (Goldschmied) Multidisciplinary Brain Research Center, Bar-Ilan UniversityRamat Gan, Israel; ^2^Research Institute for Neuroscience, Education and Didactics, Patrizio Paoletti FoundationItaly; ^3^Department of Neurobiology, Weizmann Institute of ScienceRehovot, Israel; ^4^Department of Criminology, Bar-Ilan UniversityRamat Gan, Israel; ^5^Department of Psychology, Bar-Ilan UniversityRamat Gan, Israel

**Keywords:** motor training, reflectivity, spatial cognition, EEG coherence, gender

## Abstract

Quadrato Motor Training (QMT) is a specifically-structured walking meditation, aimed at improving reflectivity and lowering habitual thought and movement. Here we set out to examine the possible effect of QMT on reflectivity, employing the Hidden Figures Test (HFT), which assesses both spatial performance (measured by correct answers) as well as reflectivity (interpolated from correct answers and reaction time). In the first study (*n* = 24, only females), we showed that QMT significantly improves HFT performance, compared to two groups, controlling for cognitive or motor aspects of the QMT: *Verbal Training* (identical cognitive training with verbal response) and *Simple Motor Training* (similar motor training with reduced choice requirements). These results show that QMT improves HFT performance above the pre-post expected learning. In the second study, building on previous literature showing gender-dependent effects on cognitive performance, we conducted a preliminary pilot examining gender-dependent effect of training on reflectivity and its electrophysiological counterparts. EEG analyses focused on theta, alpha and gamma coherence. HFT performance and resting-state EEG were measured in 37 participants (20 males), using a within-subject pre-post design. Following training, HFT performance improved in both genders. However, we found a gender-dependent difference in functional connectivity: while theta and alpha intra-hemispheric coherence was enhanced in females, the opposite pattern was found in males. These results are discussed in relation to neuronal efficiency theory. Together, the results demonstrate that QMT improves spatial performance, and may involve a gender-dependent electrophysiological effect. This study emphasizes both the importance of studying gender-related training effects within the contemplative neuroscience endeavor, as well as the need to widen its scope toward including “contemplation in action.”

## Introduction

Reflectivity is the capacity of humans to exercise introspection, by examining one's conscious thoughts and feelings, resulting in the inhibition of habitual thought or behavior. Quadrato Motor Training (QMT) is a specifically-structured walking meditation, aimed at improving reflectivity. The QMT requires a state of enhanced attention, as it combines dividing attention to the motor response and cognitive processing for producing the correct direction of movement to the next point in the Quadrato space (Paoletti and Salvagio, 2011; Dotan Ben-Soussan et al., 2013). Due to the increased awareness to the body and its location in space, in response to very specific instructions, QMT can be conceived of as “Mindful movement.” Mindful movement is a general term for practices that involve bringing awareness to the detailed experience of movement, such as when practicing walking meditation or yoga (Kabat-Zinn, 2009). Yet, in comparison to other Mindful movement practices, the QMT has the advantage of being a relatively short training (possibly several minutes), and can be relatively easily practiced in limited spaces. These unique aspects render the QMT a technique warranted of scientific exploration, with the future aim of implementing this technique in various health promoting and educational setups.

Reflectivity can be directly measured by a spatial task assessing Field Dependence-Independence (FDI) (Glicksohn and Kinberg, 2009), named the Hidden Figures Test (HFT). Here, we set out to investigate a possible QMT-induced increase in reflectivity using the HFT. We additionally utilized electroencephalography (EEG), in the attempt to relate changes in reflectivity with functional connectivity, measured by coherence, while taking into consideration gender effects for both measures (Jausovec and Jausovec, 2005; Aliakbari and Tazik, 2011). This study is referred to as Study 2, subsequently. However, as HFT performance is considered to improve following learning (Witkin et al., 1971a,b; Stericker and LeVesconte, 1982; Woodfield, 1984; Ludwig and Lachnit, 2004), it is hard to disentangle the QMT-induced improvement in HFT performance from the regular learning curve. To this end, we report the results of a study, referred to hereafter as Study 1, where HFT performance was tested in a pre-post design in a QMT group compared to two control groups, showing that QMT significantly enhanced HFT performance, well above the other groups which showed no significant change.

The construct of reflectivity has been conceptualized as a tendency to gather more information, more carefully and systematically, compared to impulsive performers (Messer, 1976). Reflective individuals are suggested to implement an analytic process and are considered more cognitively mature compared to impulsive individuals (Rozencwajg and Corroyer, 2005). Importantly, reflectivity has been found to be modifiable by training (Messer, 1976). One way to measure reflectivity is by means of a spatial task of embedded figures, originally designed to assess FDI (Glicksohn and Kinberg, 2009). FDI describes two contrasting ways of processing information, Field Dependence (FD) and Field Independence (FI), this being the most studied cognitive style (Witkin et al., 1977), and is the mode by which learners approach, acquire and process information (Witkin and Goodenough, 1981). Individuals located toward the FD end have difficulty in separating incoming information from its contextual surroundings, and are more likely to be influenced by external cues and to be non-selective in their information uptake. In contrast, FI individuals have less difficulty in separating the essential information from its context, and are more likely to be influenced by internal than external cues, and to be more selective in their information input (Riding and Cheema, 1991; Zhang, 2004). Glicksohn and Kinberg (2009) examined individual performance on embedded figures tests. Using reaction time (RT) and the number of correct detections, they postulated four templates of performance, indicative of: (1) FI—participants detect more embedded figures, and do so quite quickly; (2) FD—participants detect fewer embedded figures, and do so quite slowly; (3) impulsiveness (Imp)—individuals detect fewer embedded figures, and do so quite quickly; and (4) reflectiveness (Ref)—individuals detect more embedded figures, and do so quite slowly. It should be noted here that reflective individuals are significantly more FI than impulsive ones (Messer, 1976), and that both of these groups (FI and Ref) are thought to exhibit better performance in learning and memory tasks (Blackman and Goldstein, 1982). Here, we intend to adopt such a finely-tuned approach to the study of possible QMT-induced reflectivity, profiling individual differences. It should be considered, however, that participants tend to generally perform better on embedded figures tests after practice (Witkin et al., 1971a,b; Stericker and LeVesconte, 1982; Woodfield, 1984; Ludwig and Lachnit, 2004), hence such an improvement does not necessarily indicate an increase in reflectivity. However, within such improvement, four patterns may be discerned (Kepner and Neimark, 1984): (1) stable FI, namely high scores (number of figures detected) at both test and retest; (2) stable FD, namely low scores at both times; (3) improvement from test to retest, including (but not necessarily) a move from FD to FI; and (4) a decline in performance. Hereafter, we consider pattern 3 to operationalize an increase in reflectivity.

Depraz et al. (2000) have described the subtle dynamic of becoming more reflective in three interdependent phases: first, *suspension* from the habitual act of mind and body, then redirection of *attention* inwardly, and finally *receptivity* toward the experience. Contemplative practices can be expected to increase reflectivity, based on two arguments. First, various contemplative practices entail an improvement in attention (Brefczynski-Lewis et al., 2007; Chambers et al., 2008; Lutz et al., 2008, 2009). Second, contemplative practice, through the three phases of reflectivity (suspension, a shift of attention inwards and receptivity), enables one to disrupt normal cognitive and perceptual functioning, and hence to become more FI or reflective (Depraz et al., 2000; So and Orme-Johnson, 2001). However, there is surprisingly little research on contemplation-improved reflectivity, most of it measuring directly only the related measure of FI, and reporting conflicting results. While some studies have not found significant changes (Kurie and Mordkoff, 1970; Goldman et al., 1979), others report FI to increase following meditation (Linden, 1973; So and Orme-Johnson, 2001). Here, we study directly both reflectivity and FI. As voluntary control and planning require reflective consciousness (Legrand, 2007), we hypothesize that the QMT will induce an on-going suspension of the habitual act of moving, resulting in increased reflectivity and FI.

Meditation practices have been consistently related in EEG studies to increased power and coherence within the theta (4–7 Hz) and alpha (8–13 Hz) frequencies (Alexander et al., 1987; Aftanas and Golocheikine, 2001; Travis, 2001; Travis et al., 2002, 2009; Baijal and Srinivasan, 2010). Alpha activity increases were suggested to reflect increased relaxation; while theta increased activity has been related to heightened attention, decreased anxiety and low thought content (reviewed by Cahn and Polich, 2006). In contrast, the results relating to gamma activity (>25 Hz) have been scarce and much less consistent, some reporting increased gamma activity (Lutz et al., 2004) while others reporting decreased activity (Faber et al., 2004; Berkovich-Ohana et al., 2012, 2013; Lehmann et al., 2012). While meditation research examined both power and functional connectivity, research examining whole-body contemplative movement practices, such as Tai Chi and Qigong, has mostly focused on power, reporting increased frontal theta and alpha activity (reviewed by Cahn and Polich, 2006). As EEG coherence changes in the context of contemplative practices are usually confined within the theta, alpha, and gamma bands, our study focuses on these bands in the analyses.

Results regarding the connection between FDI and EEG coherence are confusing. While some studies found that FI participants display less inter-hemisphere alpha coherence, suggesting more hemispheric specialization (O'Connor and Shaw, 1978; Oltman et al., 1979; Zoccolotti, 1982), others have found that FI participants had higher alpha coherence compared to those who were FD (Colter and Shaw, 1982). Nevertheless, we hypothesized that our participants should exhibit more reflectivity and FI, hence an increase in inter-and intra-hemispheric EEG coherence, especially within the alpha and theta bands (Colter and Shaw, 1982). Gender may, however, moderate this, given that: (1) baseline EEG coherence is higher in females than in males (van Beijsterveldt et al., 1998), though this might be reversed in the gamma band (Jausovec and Jausovec, 2010); (2) an increase in both alpha and theta coherence from baseline to task is more prominent in females than in males (Beaumont et al., 1978; Volf and Razumnikova, 1999).

It is important to consider that gender-dependent differences have been frequently observed in both the motor and the cognitive realms (e.g., Kimura, 1992; for review, see Diamond et al., 1983; Baron-Cohen and Hammer, 1997). The different gender-dependent functional connectivity effects have been related to gender differences in brain structures, especially within the corpus callosum (Corsi-Cabrera et al., 1997; Jausovec and Jausovec, 2005). Hence, in the first study we studied only females to reduce intra-group variability. In the first study, we set out to study the effect of QMT on HFT performance by comparing the QMT to two control groups: *Verbal Training* (VT, identical cognitive training with verbal response) and *Simple Motor Training* (SMT, similar motor training with reduced choice requirements). As will be shown, results indicated that only the QMT group, and not the control groups, showed better HFT performance. In the second study we set out to examine gender-dependent differences, comparing the effects of QMT for male and female participants on reflectivity, as well as studying the possible EEG counterparts. As will be shown, both genders exhibited better HFT performance and reflectivity following the QMT, albeit showing an opposite EEG pattern. These results are discussed in light of neuronal efficiency theory.

## Study 1

### Methods

#### Participants and design

A total of 24 female (28 ± 3 years in age) students participated in the study, none of whom practiced QMT before (for the detailed procedure, see Dotan Ben-Soussan et al., 2013). In order to avoid gender-dependent differences in performance (Terlecki et al., 2008) and reduce intra-group variability we focused on female participants. Data were collected both before and after a single training session lasting 7 min in each of three training groups (the participants having been randomly allocated to these): (1) Quadrato Motor training (QMT—3 choices and whole-body response, *n* = 9); (2) Simple motor training (SMT—1 choice and whole-body response, *n* = 7); and (3) Verbal training (VT—3 choices and verbal response, *n* = 8). The collected data included the HFT, reported here. Other measures included EEG and an Alternative Uses task, reported elsewhere (Dotan Ben-Soussan et al., 2013).

#### Training groups

**Figure 1:**
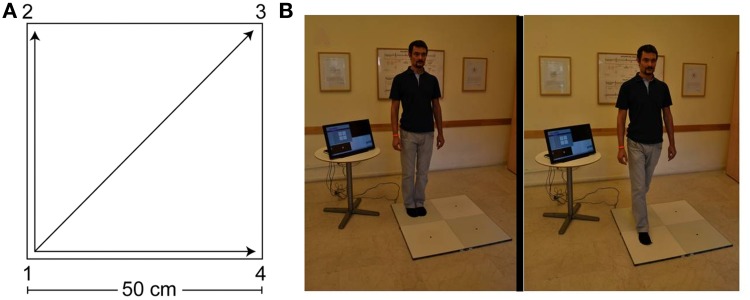
**The Quadrato Motor Training (QMT). (A)** A graphical illustration of the QMT. **(B)** A participant during the QMT while waiting for the next instruction (left) and following the instruction (right).

***Quadrato Motor Training (QMT)***. Briefly, the participant stood at one corner of a 0.5 m × 0.5 m square and made movements in response to verbal instructions given by an audio tape recording. There were three optional directions of movement. The instructions directed participants to keep the eyes focused straight ahead, hands loose at the side of the body. They were also told to immediately continue with the next instruction and not to stop due to mistakes. At each corner, there were three possible directions to move. The training thus consisted of 12 possible movements (Figure 1). Training consisted of a sequence of 69 commands, lasting 7 min.

Two variables that were addressed in other studies of motor learning are limb velocity and the decision regarding the responding limb (Criscimagna-Hemminger et al., 2002; Donchin et al., 2003). In order to control these parameters, we used a movement sequence paced at a rate of an average of 0.5 Hz (similar to a slow walking rate), and we instructed the participants to begin all movements with the leg closest to the center of the square (a detailed description can be found in Dotan Ben-Soussan et al., 2013).

***Simple Motor Training (SMT)***. The SMT group provided similar motor performance as the QMT group but with reduced cognitive demands. This group moved from corner to corner on the square in exactly the same manner as the QMT group (pace, duration, auditory cue), but their movement was consistently 1-2-3-4-1 etc. The participants heard the same recordings as the QMT group. However, while the QMT group was told that each number represented a different corner of the square, the SMT group was told to simply begin at a certain corner and to continue to the next corner clockwise in response to the instructions. That is, regardless of the number specified on the tape, they always moved in the same sequence. This reduced the uncertainty and the cognitive demand, compared to the QMT group.

***Verbal Training (VT)***. The VT group was designed to reduce motor load while keeping the same cognitive load and uncertainty. The participants, who were instructed to only make verbal responses, stood 1 m in front of the square, but did not move to the corners. Instead, they responded to the taped commands verbally by stating what direction of movement would be required in order to reach the corner specified by the command. For example, for a movement from corner 1 to corner 2, they were required to say “straight.” All other training parameters were kept identical to the QMT (pace, duration, auditory cue).

#### Hidden figures test

**Figure 2:**
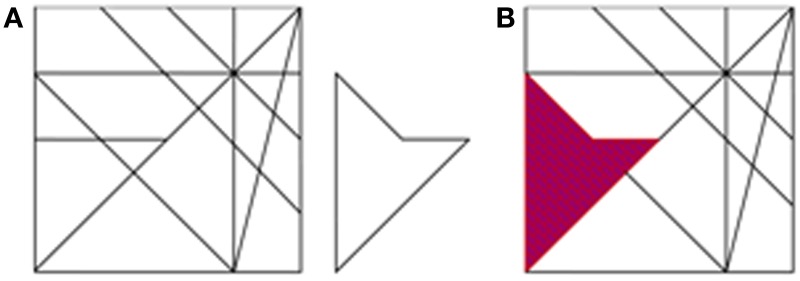
**The Hidden Figures Task (HFT). (A)** An example of one trial. **(B)** The correct response for trial A (purple).

We employed a computerized version of the Hidden Figures Test (HFT, detailed in Glicksohn and Kinberg, 2009), generated using E-Prime 1.1 (Psychology Software Tools). The participants were required to locate a simple figure embedded within a complex figure, both of them appearing on the screen, side by side (Figure 2). The figures ranged in size between 10.5 × 4 cm and 12 × 6.5 cm, and were viewed from a comfortable viewing distance. The participants were allocated 30 s for each of 16 trials. Based on the data collected by Glicksohn and Kinberg (2009), we split the test into two even sets of 8 tasks, matched for degree of difficulty, defined as number of hits for item/(number of hits + number of false alarms for item) in that study (*n* = 80). The two sets were presented in a counterbalanced order across participants. Before the test, one practice trial was given. The participant attempted to locate the simple figure as quickly as possible, within the time allocated. On detecting the figure, the participant pressed a button, and the complex figure was presented again, so that the participant could indicate the embedded figure for the experimenter. Two scores were obtained for each trial: correct/incorrect detection, and reaction time (RT). All RTs were subsequently log-transformed to normalize the data, and mean log(RT) was computed for each participant.

#### Statistical analyses

To answer the question regarding the effects of QMT on HFT performance, we ran a Group (QMT, SMT, VT) × Training (pre, post) analysis of variance (ANOVA) for correct HFT responses, adopting the Greenhouse-Geisser criterion. Whenever needed, we added *post-hoc t*-tests.

### Results

**Figure 3:**
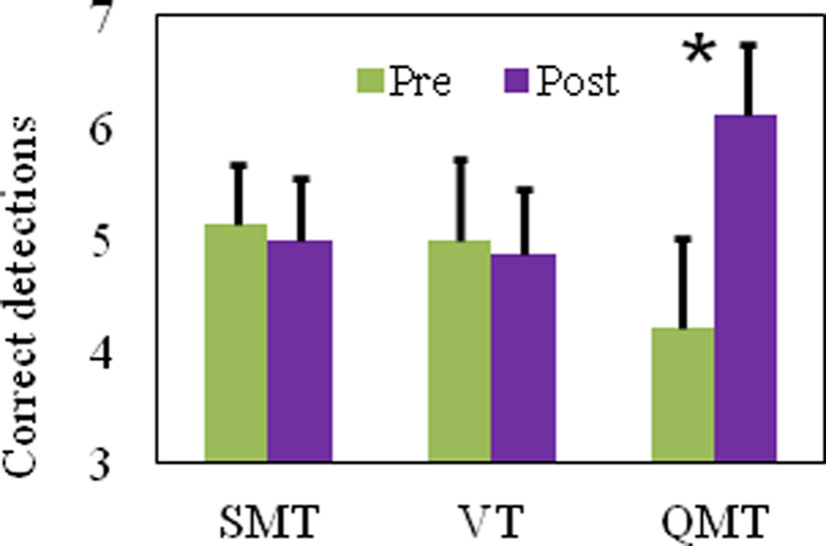
Number of correct detections in the HFT task as a function of Group and Training (mean ± SEM, *p < 0.05).

We report a significant Group × Training interaction [*F*_(2, 21)_ = 5.95, *MSE* = 0.63, *p* < 0.01] for the number of correct detections in the HFT. For QMT, correct detections significantly increased [*t*_(8)_ = −2.86, *p* < 0.05] in contrast to SMT and VT which showed no change following training (see Figure 3).

To summarize Study 1, the QMT-induced improved performance on the HFT was significantly higher compared to the two control groups, which emphasized either the cognitive or the motor training of QMT, but not both. The two control groups showed no improvement, indicating that in this setup there is no significant improvement due to the pre-post HFT learning itself. Subsequently, in Study 2, as a continuation of Study 1, we wanted to examine whether similar effects could be observed in male participants following QMT.

## Study 2

### Methods

#### Participants and design

A total of 37 volunteers (29 ± 4 years in age) participated in the study, which was conducted both in Israel (10 females—out of which nine participated in Study 1 in the QMT group, and 8 males) in the MEG unit at the Gonda Brain Research Center, and in Italy (7 females, and 12 males) in the cognitive neurophysiology laboratory of the Research Institute for Neuroscience Education and Didactics. All participants were right-handed with no medical history that might affect their EEG. The study was approved by the ethics committee of Bar-Ilan University. Upon entering the lab, the participant signed a written informed consent. Then, we recorded baseline EEG for 5 min (2.5 min eyes open and fixed and then 2.5 min eyes closed). Subsequently, the HFT Task (see section Hidden Figures Test) was presented. All data were collected both before and after a single QMT session lasting 7 min. In order to pool the female participants together and all male participants together, we conducted two *t*-tests (the first for comparing the participants who took part in Study 1 to the additional 8 females; and the second for comparing the Israeli and Italian male participants). The two independent *t*-tests revealed no differences in performance in the HFT within the female and male groups, allowing us to pool our participants.

#### Hidden figures test

The task was the same as in Study 1. In addition to the analysis conducted as in section Hidden Figures Test, based on Glicksohn and Kinberg (2009), four profiles were defined: (1) Field Dependent (FD) = low success + long RT; (2) Field Independent (FI) = high success + short RT; (3) Reflective (Ref) = high success + long RT; (4) Impulsive (Imp) = low success + short RT. High success was defined as an above-median number of correct detections at baseline (median = 4); long RT was defined as an above-median Log(RT) at baseline (median = 4.21).

#### Electrophysiological measurements

EEG data were recorded using a 65-channel geodesic sensor net (Electrical Geodesics Inc., Eugene, USA), sampled at 500 Hz and referenced to the vertex (Cz) with analog 0.1–200 Hz band-pass filtering. Impedance was usually kept under 40 kΩ, lower than the customary 50 kΩ with this system (Ferree et al., 2001). EEG signals showing eye movements or muscular artifacts were manually excluded, and bad channels were replaced using spatial interpolation (Perrin et al., 1989). The data were referenced offline to average reference. The first 32 non-overlapping, artifact-free epochs of 2.048 s duration were extracted from each electrode for further analysis from the eyes-closed resting state period, as previously reported (Dotan Ben-Soussan et al., 2013). Coherence values were calculated from the multitapered pair-wise cross-spectra. The coherence values were normalized using Fisher's *z* transformation. We calculated coherence within the theta, alpha, and gamma frequencies (4–7, 8–13, 25–45 Hz, respectively). Notably, gamma effects are at risk of contamination by muscle activity from scalp and neck (Whitham et al., 2007) and saccade-related spike potentials (SP) due to eye movements (Yuval-Greenberg and Deouell, 2009). In order to minimize this risk, and as SP is elicited at the onset of small saccades, which occur during eyes-open fixation (Martinez-Conde et al., 2004), our report focuses on eyes-closed conditions. Furthermore, to eliminate muscle artifacts, we used three additional steps for caution. First, we carefully visually inspected the raw data, manually extracting artifact-free epochs. Second, as the EMG peaks at 70–80 Hz (Cacioppo et al., 1990), we used much lower frequencies of 25–45 Hz. And third, we excluded from statistical analyses all the circumference electrodes, closest to eyes, neck and face muscles (Berkovich-Ohana et al., 2012).

**Figure 4:**
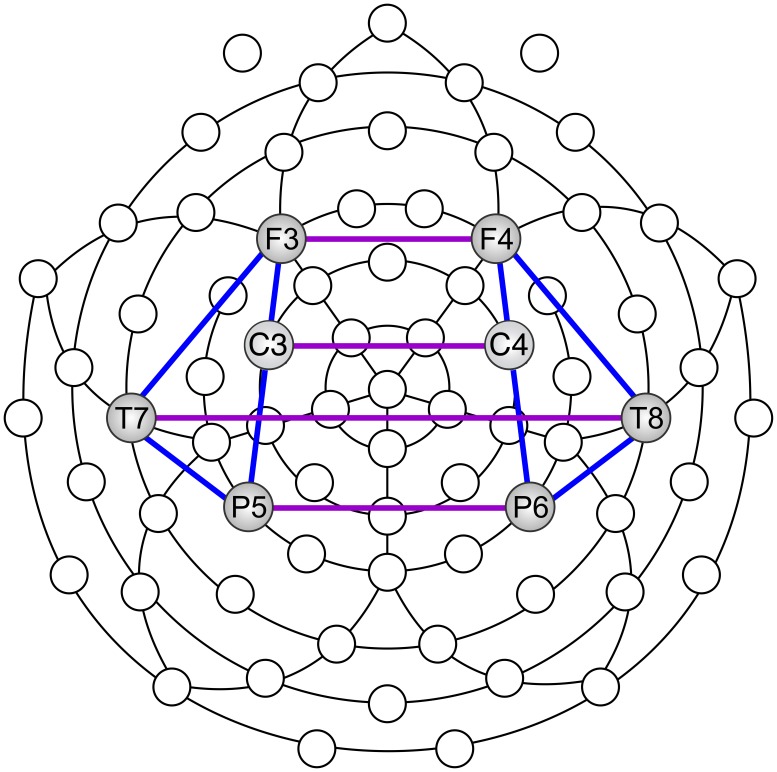
**Scalp locations used to calculate EEG coherence: inter-hemispheric (purple) and intra-hemispheric (blue)**.

For the sake of data reduction and statistical comparisons, we chose one central electrode site in each region of interest. Since frontal, central, temporal and parietal areas are important for cognition and action, we chose to focus on bilateral frontal, central, temporal, and parietal electrode sites (F3, F4, C3, C4, T7, T8, P5, P6) (Figure 4). We defined electrode “pairs of interest” (POI), on the basis of prior knowledge concerning movement and meditation (Andres et al., 1999; Lutz et al., 2004, 2007).

#### Statistical analyses

In order to answer the question of gender-difference in HFT performance, we ran a Gender × Training (pre, post) analysis of variance (ANOVA) for the two measures of HFT: (1) number of correct detections; (2) mean log(RT). Whenever needed, we added *post-hoc* one-tailed *t*-tests. Change in number of correct detections was calculated by subtracting the number of correct figures detected before QMT from the number of correct detections following QMT. Change in log(RT) was calculated by subtracting pre from post QMT log(RT).

Then, in order to study the possible gender-dependent electrophysiological counterparts of the QMT effects, we ran six ANOVAs for the *z*-transformed theta, alpha and gamma coherences, for intra- and inter-hemispheric connections separately. For intra-hemispheric coherence, we ran a 4-way ANOVA: Gender × Training × Hemisphere × Electrode Pair (F-T, F-P, T-P). For inter-hemispheric coherence, we ran a Three-Way ANOVA: Gender × Training × Bilateral Electrode Pair (F-F, C-C, T-T, P-P). For all the ANOVAs we adopted the Greenhouse-Geisser criterion.

### Results

#### HFT

**Figure 5:**
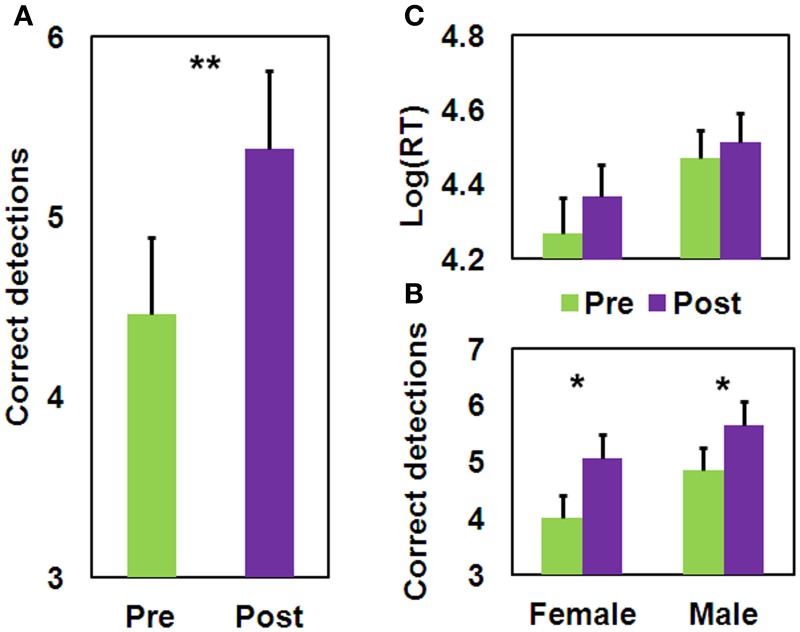
**Pre-post difference in performance on the HFT task (mean ± SEM). (A)** Main effect for pre-post difference in number of correct detections; **(B)** Pre-post difference in number of correct detections as a function of Gender; **(C)** Pre-post difference in mean log(RT) as a function of Gender; (^*^p < 0.05; ^**^p < 0.005).

**Figure 6:**
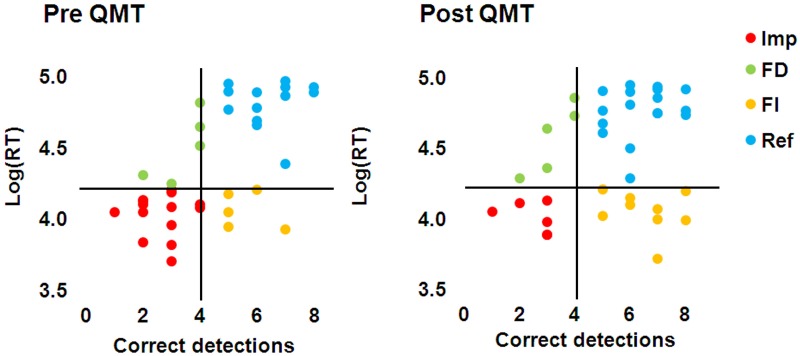
**HFT performance and RT correlation pre and post QMT**. Quadrants show grouping of participants into Impulsive (Imp), Field Dependent (FD), Field Independent (FI), and Reflective (Ref) groups.

We found a significant Training effect [*F*_(1, 35)_ = 9.57, *MSE* = 1.66, *p* <.005] for the number of correct detections (Figure 5A), which extend the results of Study 1 to males; but not for mean log(RT). The Gender × Training interaction was not significant for either measure. No difference in performance between the male and female participants was found either at baseline, or following training, for either measure (Figures 5B,C). As can be seen from Figure 5, on average there is an increase in the detection of one more figure following QMT, and the test-retest correlation found here of 0.57 (*n* = 37, *p* < 0.0001) is relatively high, though is lower than the.78-.92 range found for the GEFT (Kepner and Neimark, 1984). This correlation is comparable to the correlation that we have computed between the two sets derived from the HFT of a previous study (Glicksohn and Kinberg, 2009), which is.67 (*n* = 80, *p* < 0.0001). An overall view of the relationship between HFT performance (see section Results) and RT is presented as a scatter plot (Figure 6). Of the 37 participants, 12 (6 females) exhibited a trend toward reflectivity or FI following QMT. The results show increased reflectivity in both genders.

A positive correlation was found between the change in score for the two change in number of correct detections and change in log(RT) measures (*r* = 0.36, *p* < 0.05, *n* = 37), indicating that better performance on the HFT following QMT is related to longer latency of correct response. Again, we can compare this to the respective difference scores computed for two sets derived from the HFT of the previous study, which is 0.22 (*n* = 76, *p* = 0.053). Thus, it is not simply the case that QMT leads to *faster* RT, as reported elsewhere for a simple reaction time task (Dotan Ben-Soussan et al., 2013), rather that performance on the HFT is indicative of reflectiveness (more correct detections coupled with longer RT; Glicksohn and Kinberg, 2009).

#### Electrophysiological data

**Figure 7:**
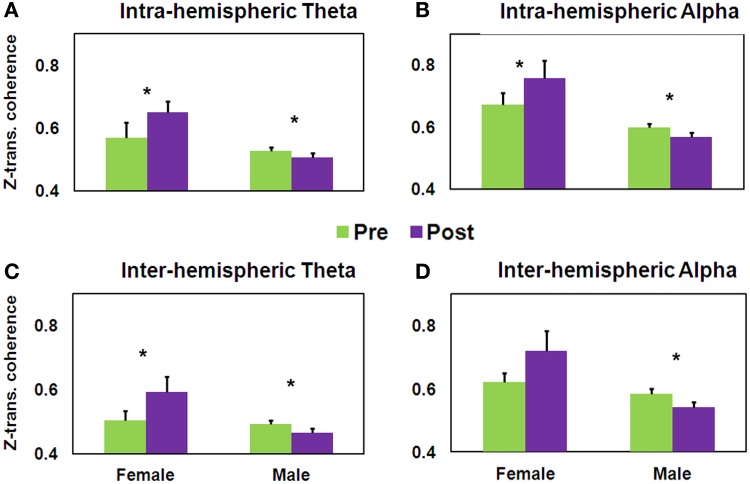
**Pre-post differences in *z*-transformed coherence as a function of Gender**. Intra-hemispheric coherence for **(A)** theta and **(B)** alpha frequency bands; and inter-hemispheric coherence for **(C)** theta and **(D)** alpha frequency bands (mean ± SEM, ^*^p < 0.05). Note: these are coherence and not squared coherence values.

Of importance to the question related to training-induced gender-dependent effects, the first set of three ANOVAs yielded within the theta band a significant Gender × Training interaction [*F*_(1, 35)_ = 11.32, *MSE* = 0.03, *p* < 0.005]. As seen in Figure 7A, while the female group demonstrated significantly increased intra-hemispheric theta coherence [*t*_(16)_ = −2.56, *p* < 0.05], this was significantly decreased in the male group [*t*_(19)_ = 2.57, *p* < 0.05]. A similar pattern was found within the alpha band, the Gender × Training interaction being significant [*F*_(1, 35)_ = 7.22, *MSE* = 0.05, *p* < 0.05]. As seen in Figure 7B, while the female group demonstrated significantly increased intra-hemispheric alpha coherence [*t*_(16)_ = −2.02, *p* < 0.05], this was significantly decreased in the male group [*t*_(19)_ = 1.76, *p* < 0.05]. No such interaction was found for intra-hemispheric gamma coherence.

The second set of three ANOVAs yielded a significant Gender × Training interaction for both theta [*F*_(1, 35)_ = 11.05, *MSE* = 0.02, *p* < 0.005] and alpha coherence [*F*_(1, 35)_ = 5.92, *MSE* = 0.06, *p* < 0.05]. As seen in Figures 7C,D, while the female group demonstrated significantly increased inter-hemispheric theta coherence [*t*_(16)_ = −2.46, *p* < 0.05], theta and alpha coherence significantly decreased in the male group [*t*_(19)_ = 2.74, 1.81, respectively, *p* < 0.05]. *Post-hoc* analysis revealed a significant increase in temporal alpha coherence in the females who did not improve having low HFT performance at baseline [*t*_(4)_ = −4.87, *p* < 0.01], while an opposite pattern was observed in males. More specifically, frontal alpha coherence significantly decreased in the males who improved [*t*_(6)_ = 2.84, *p* < 0.05]. Although not significant, a similar trend occurred for intra-hemispheric alpha. In addition, a significant positive correlation was found between change in theta and alpha coherence, both for intra- and inter-hemispheric connections (*r* = 0.75, and 0.79, respectively, *p* < 0.0001, *n* = 37), in accordance with previous reports (Sauseng et al., 2002; van Albada and Robinson, 2013).

**Figure 8:**
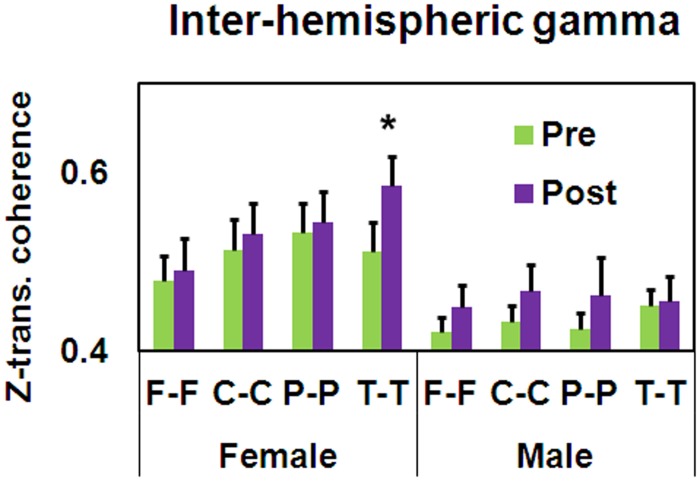
Pre-post differences in *z*-transformed gamma coherence as a function of Gender and Electrode Pair (mean ± SEM, ^*^p < 0.05).

In addition, a significant Gender × Training × Electrode Pair interaction was found for inter-hemispheric gamma coherence [*F*_(3, 105)_ = 3.01, *MSE* = 0.01, *p* < 0.05]. As seen in Figure 8, only the female group demonstrated post-training increased inter-hemispheric bilateral temporal gamma coherence [*t*_(16)_ = −2.16, *p* < 0.05].

Marginally significant differences were found between the female and male groups at baseline for inter- and intra-hemispheric gamma coherence [*t*_(35)_ = 1.97, 1.96, *p* = 0.06], but not for theta or alpha. Following QMT, a significant difference was found for intra-hemispheric theta and alpha [*t*_(35)_ = 2.80, 2.79, *p* = 0.008]. A similar difference was found for inter-hemispheric theta and alpha [*t*_(35)_ = 2.36, 3.38, *p* < 0.05] but not for gamma coherence.

To summarize Study 2, following training, reflectivity improved for 32% of the participants. Importantly, while a similar trend of improved reflectivity was observed in both genders, there was a differential effect in EEG coherence for males and females. More specifically, temporal alpha increased in the females, while an opposite pattern was observed in males. In addition, the female group demonstrated post-training increased inter-hemispheric bilateral temporal gamma coherence

## Discussion

### QMT improves reflectivity

The behavioral result of Study 1 has demonstrated that a session of QMT improved performance in the HFT task. This was in contrast to SMT and VT, representing the motor and cognitive aspects of the training, respectively. In addition, Study 2 indicates that training enabled our participants to become more reflective, as well as more FI. The current results suggest that a short practice (7 min) of QMT can be advantageous for improving reflectivity. This supports the claim raised by Depraz et al. (2000) that although reflectivity can sometimes be thought to require a long duration of time to manifest itself, it might be triggered faster, especially in situations we are not accustomed to in our daily life. The current results strengthen previous claims that simple cognitive tests measuring solely reaction time are not sufficiently sensitive to evaluate changes in higher cognitive functions (Dietrich and Sparling, 2004; Dotan Ben-Soussan et al., 2013) such as reflectivity.

What could be the cause of the QMT-improved reflectivity? The three inter-dependent phases described for the dynamics of a reflective act include suspension, redirection of attention inwardly and receptivity, with suspension of habitual activity being the most important to initiate the reflective act (Depraz et al., 2000). In relation to that, it could be argued that given the continual state of attending and waiting for the next instruction during the QMT, the participants were obliged to enter this state of *suspending* the tendency for habitual movement, that of moving where and when you want. In fact, the QMT requires re-instantaneous suspended acts. The suspended movement that begins the process of reflectivity is “forced” here by a different instruction at each step of the QMT. That is, the relatively long inter stimulus intervals (1200 ms) between current location and next location, as well as waiting within the position until the next instruction in the QMT can be regarded as empty time, a time of silence. However, even when it is very brief, a silence of a few seconds can appear exponentially longer (Depraz et al., 2000). Pockett (2003, p. 63) has suggested that when one is “actively engaged in the external world” (as these participants had to be) then “time slows down.” We thus suggest that it is this bodily awareness required in the QMT combined with attending that reduce reactivity and increase reflectivity (Depraz et al., 2000; Bishop et al., 2004; Kirk et al., 2011; Greenberg et al., 2012). Furthermore, the QMT requires dividing *attention* inwardly and outwardly, simultaneously listening to the instruction as well as attending to the position of the body in space. In line with that, Depraz et al. (2000) have related to the inner/outer distinction, claiming that the time of relative emptiness (represented here as the time with no specific instructions except the attending, waiting for the next instruction) enables the transition from “looking for” to “letting come,” which in turn leads to and further requires a state of receptiveness, which in turn removes the inside/outside distinction (Depraz et al., 2000), and creates a “space for silence” related to increased reflectivity and expansion of time (Dawson, 2003; Paoletti and Salvagio, 2011).

In support of this claim, it was previously shown that another form of contemplative practice, Mindfulness meditation, dilates the subjective time experienced, in comparison to control participants (Berkovich-Ohana et al., 2012). Indeed, accumulating findings show that Mindfulness brings one back to greater awareness to the present moment (Farb et al., 2007; Kabat-Zinn, 2009; Brewer et al., 2011; Berkovich-Ohana et al., 2012; Vago and Silbersweig, 2012) and to the body (Farb et al., 2007, 2012; Price and Thompson, 2007; Kerr et al., 2013). Possibly, similar to Mindfulness, QMT increases attention to the body and to the present moment by requiring attendance to the coming instruction and consequently responding to it. This in turn enables a state of increased reflectivity.

It could be suggested that increased reflectivity was only for one third of the participants that we report due to the short training. Possibly, a longer session, or a longer-term training with similar short sessions might result in increased percentages of enhanced reflectivity. However, these hypotheses can only be studied in a follow-up study.

### Gender-dependent electrophysiological changes

We hypothesized that better performance on the HFT would be related to increased alpha coherence, and that QMT would increase coherence, and more so in females. As it turned out, both hypotheses were supported by the results for the female group, and were opposite for the male group, hence underscoring the importance of studying gender differences.

As a starting point, we observed overall gender differences in pre-QMT resting state coherence, indicative of trait effects, with males exhibiting lower coherence (both intra- and inter-hemisphere) in the theta, alpha and gamma bands compared to females. These results are consistent with a previous study of highly intelligent individuals, reporting that males displayed greater decoupling (mostly of frontal brain areas), whereas females showed the opposite pattern, namely of more coupling (mostly between frontal and parietal areas) (Jausovec and Jausovec, 2005). This is further in line with the idea of differential brain organization in males and females (Volf and Razumnikova, 1999; Amunts et al., 2000; Mohr et al., 2005), whereby females consistently display higher inter-hemispheric coherence at rest and during cognitive task performance compared to males (Beaumont et al., 1978; Corsi-Cabrera et al., 1997; Volf and Razumnikova, 1999). The higher female coherence was previously interpreted as reflecting higher collaboration between the hemispheres or less hemispheric specialization for females (Amunts et al., 2000; Mohr et al., 2005; Ramos-Loyo and Sanchez-Loyo, 2011), suggesting that the left hemispheric is more dominant for different abilities, such as language and navigation in males, in comparison to females (Volf and Razumnikova, 1999; Ramos-Loyo and Sanchez-Loyo, 2011). The decreased trait coherence observed in the male group in the current study may thus be related to increased hemispheric specialization in males in general.

#### Training-related gender differences

Showing a similar pattern as the differential gender trait differences, QMT-induced electrophysiological changes included significantly increased theta and alpha coherence in females, in support of our hypothesis, while the opposite was found for males. The increased theta and alpha coherence observed in the female group is consistent with studies examining electrophysiological changes following meditation. Meditative practices, irrespective of gender, have consistently been related to increased coherence, especially inter- and intra-hemispheric theta and alpha coherence (Alexander et al., 1987; Aftanas and Golocheikine, 2001; Travis, 2001; Travis et al., 2002, 2009; Cahn and Polich, 2006; Baijal and Srinivasan, 2010). Unfortunately, gender-related analyses within meditation reports are scarce, as either gender is not reported at all (e.g., Lutz et al., 2004), or there are no gender-specific baseline differences, hence these are not subsequently considered (Travis et al., 2009), or not mentioned (e.g., Aftanas and Golocheikine, 2001; Travis et al., 2002; Baijal and Srinivasan, 2010). Although gender differences have not yet been systematically investigated in previous electrophysiologically-based meditation studies, some gender differences in behavioral measures were found following meditation. For example, while Qigong meditation was found to contribute positively to addiction treatment outcomes in both genders, female participants reported significantly more reduction in anxiety and withdrawal symptoms than did the male group (Chen et al., 2010). An opposite pattern was observed for TM, for which reduced drinking rates was reported among male university students but not in female students (Haaga et al., 2011). Our finding of QMT-induced gender-related differences in coherence is in accord with another study reporting gender-related electrophysiological differences. In this study (Duregger et al., 2007), a motor related evoked potential (contingent negative variation—CNV) was shown to manifest differently in females, showing higher frontal activation, compared to males, showing higher temporoparietal activation. The more frontal brain area was related to motor preparation reflecting a higher level of cognitive involvement in females compared to males, while the higher male temporoparietal brain activity was interpreted as reflecting brain processes related to other sensory processing (Duregger et al., 2007). Related to this, it is important to keep in mind the gender-dependent differences frequently observed in motor and cognitive realms (Baron-Cohen and Hammer, 1997). More specifically, while women are better in fine-motor coordination (e.g., placing pegs in pegboard holes) men are better in target-directed motor skills, such as guiding or intercepting projectiles (Kimura, 1992). Finally, there are gender differences in brain morphology in relation to spatial cognition and mental rotation performance: while females have more gray matter in the parietal lobe than males, males have greater parietal surface area (Koscik et al., 2009). Koscik et al. (2009) further concluded that the structural gender difference could be a neurobiological substrate for the gender difference in mental rotation performance. In light of the above studies, showing gender-dependent strategies and neural structure, the current results of gender-dependent electrophysiological change following training could be interpreted as reflecting different strategies to achieve the same cognitive and motor goal.

The gender-dependent results may further be explained by the neuronal efficiency hypothesis, which claims that better performance is associated with more efficient brain functioning, that is to say, decreased cortical activation (Haier et al., 1988), indicated by lower event-related desynchronization (ERD). This hypothesis was examined in different tasks, such as spatial cognition (Neubauer et al., 2002; Grabner et al., 2006). One study examining gender-dependent changes in neuronal efficiency and the effects of training on a mental rotation task (Neubauer et al., 2010), reported that while a general increase in neural efficiency from pre to post-test was found for males, it was observed in females only in the 3D version and not in the 2D presentation of the task. In addition, previous studies have also shown that males and females display neuronal efficiency and an inverse IQ–activation relationship in just that domain in which they usually perform better, i.e., females in the verbal domain, and males in the visuospatial domain (Neubauer et al., 2005). Important to the current results, it was further suggested that neural efficiency in males is reflected in local cortical activation (as measured with the ERD-method), whereas neural efficiency in females is manifested in the functional coupling of several brain areas, as assessed by EEG coherence (Neubauer and Fink, 2003). Following this line of thought, training should lead to an increase in neural efficiency, namely, to a decrease in cortical activation from pre- to post-test (Kelly and Garavan, 2005; Neubauer and Fink, 2009; Neubauer et al., 2010) in a gender-dependent manner. Thus, the QMT-induced electrophysiological changes may amplify resting state gender trait differences.

### Limitations of the study

It should be kept in mind that Study 2 is a preliminary attempt to examine the gender-dependent effects of training. Although Study 2 may be confounded by practice, in Study 1 as well as in previous research (Dotan Ben-Soussan et al., 2013), we have demonstrated that the cognitive change, namely improved spatial cognition and creativity, is QMT-specific, and does not occur in the two control groups. In addition, we have previously demonstrated a QMT-specific effect on EEG coherence, in contrast to the two control groups (Dotan Ben-Soussan et al., 2013). Nevertheless, as suggested by one of our reviewers, a future study incorporating a larger number of participants could deepen the research regarding the electrophysiological gender-dependent effects of QMT.

Obviously, a longitudinal design is warranted to elucidate the long-term effects of QMT. Furthermore, this study lacks phenomenological reports, especially valuable when studying subtle mental states such as reflectivity (Varela et al., 1991; Depraz et al., 2000). Undoubtedly, future QMT studies in our lab will incorporate qualitative in-depth interviews. Taken together, this study should be considered as an exploratory pilot study, warranting further exploration.

### Summary and possible implication

The first important finding in this report is that one short (7 min) QMT session improves spatial cognition, as opposed to the two control groups reported in Study 1. In addition, following training reflectivity improved for 32% of the participants. As QMT enhances spatial performance and may increase reflectivity, it would be worthwhile to examine it in the context of different learning and impulsivity-related disorders. Given that coherence alterations have long been observed in ADHD and dyslexia (Dhar et al., 2010; Barry et al., 2011), future studies should examine the effects of QMT in these populations. Critically, the advantage of QMT training lies in its simplicity of training, and minimal space needed compared to other movement contemplative practices, such as martial arts or walking meditation.

Our results suggest that the underlying electrophysiological mechanism by which QMT exerts its effect differs between genders. This has been discussed in light of gender-related brain specialization, as well as the neuronal efficiency hypothesis. This study emphasizes both the importance of studying gender-related training effects within the contemplative neuroscience endeavor, as well as the need to widen the scope of contemplative neuroscience toward including “contemplation in action” such as QMT.

## Author contributions

Conceived and designed the experiments: Tal Dotan Ben-Soussan, Joseph Glicksohn, Aviva Berkovich-Ohana. Performed the experiments: Tal Dotan Ben-Soussan. Analyzed the data: Tal Dotan Ben-Soussan, Aviva Berkovich-Ohana, Joseph Glicksohn. Contributed analysis tools: Tal Dotan Ben-Soussan, Aviva Berkovich-Ohana, Joseph Glicksohn, Abraham Goldstein. Wrote the paper: Tal Dotan Ben-Soussan, Aviva Berkovich-Ohana, Joseph Glicksohn, Abraham Goldstein.

### Conflict of interest statement

The authors declare that the research was conducted in the absence of any commercial or financial relationships that could be construed as a potential conflict of interest.
